# Congruent responses to weather variability in high arctic herbivores

**DOI:** 10.1098/rsbl.2012.0764

**Published:** 2012-09-26

**Authors:** Audun Stien, Rolf A. Ims, Steve D. Albon, Eva Fuglei, R. Justin Irvine, Erik Ropstad, Odd Halvorsen, Rolf Langvatn, Leif Egil Loe, Vebjørn Veiberg, Nigel G. Yoccoz

**Affiliations:** 1 Norwegian Institute for Nature Research, Fram Centre, 9296 Tromsø, Norway; 2 Norwegian Polar Institute, Fram Centre, 9296 Tromsø, Norway; 3 University of Tromsø, 9037 Tromsø, Norway; 4 The James Hutton Institute, Aberdeen AB15 8QH, UK; 5 Norwegian School of Veterinary Science, 0033 Oslo, Norway; 6 Natural History Museum, University of Oslo, 0318 Oslo, Norway; 7 Norwegian Institute for Nature Research, 7485 Trondheim, Norway; 8 University Centre in Svalbard, 9171 Longyearbyen, Norway; 9 Norwegian University of Life Sciences, 1432 Aas, Norway; 10 CEES, Institute of Biology, University of Oslo, 0316 Oslo, Norway

**Keywords:** arvicolinae, caribou, ungulate

## Abstract

Assessing the role of weather in the dynamics of wildlife populations is a pressing task in the face of rapid environmental change. Rodents and ruminants are abundant herbivore species in most Arctic ecosystems, many of which are experiencing particularly rapid climate change. Their different life-history characteristics, with the exception of their trophic position, suggest that they should show different responses to environmental variation. Here we show that the only mammalian herbivores on the Arctic islands of Svalbard, reindeer (*Rangifer tarandus*) and sibling voles (*Microtus levis*), exhibit strong synchrony in population parameters. This synchrony is due to rain-on-snow events that cause ground ice and demonstrates that climate impacts can be similarly integrated and expressed in species with highly contrasting life histories. The finding suggests that responses of wildlife populations to climate variability and change might be more consistent in Polar regions than elsewhere owing to the strength of the climate impact and the simplicity of the ecosystem.

## 1. Introduction

Assessing the role of climatic variation in the dynamics of wildlife populations has become a pressing task in the face of current rapid climate change, particularly in the Arctic [1]. However, quantifying climate impacts on populations in order to derive generally applicable predictions is often hampered by strongly nonlinear responses modified by species-specific life histories [2] and/or ecosystem complexity [3,4]. Nonetheless, the simplicity of terrestrial high Arctic ecosystems could provide evidence of consistent responses among wildlife species leading to potentially powerful predictions of the likely consequences of future climate change.

Reindeer (*Rangifer* sp.) and small rodents are abundant components of the arctic ecosystem that often show large fluctuations in population densities [5,6]. While their trophic position is similar, they are fundamentally different in most other traits. Reindeer are large-sized (60–200 kg) and long-lived (typically max. 10–20 years) with a maximum of one calf per year. By contrast, rodents are small-sized (0.03–0.2 kg) and short-lived (typically max. 1–2 years) with several large litters per year. Such differing life-history traits have been suggested to cause different responses to environmental variation [7–9]. Arctic rodents often show population cycles that are thought to be driven by predator–prey interactions or grazing-induced food quantity cycles [5], while population dynamics of reindeer in predator-free Arctic environments are in general thought to be driven by competition for food, with additional between-year variation in vital rates caused by climatic effects on the quantity and quality of food available [10–13] and/or parasite abundances [14]. A phenomenon that has the potential to affect both species is ground ice that encases the vegetation and thereby limits access to forage in winter [15,16], with effects propagating through their population dynamics [17–20], and ultimately the web of interactions these herbivores are driving in Arctic ecosystems [3,10,18,21]. Rain-on-snow (ROS) events in the Arctic result in ground ice when the rain water percolates through the snow and freezes as it meets the frozen ground [17,22], although the empirical evidence for the importance of such events has been questioned [23]. Here, we report the results from two long-term studies in the high Arctic that for the first time show evidence for strong temporal synchrony in the population dynamics of reindeer and small rodents. This synchrony is caused by climate forcing, of which ROS events is a main component.

## 2. Material and methods

On the island of Spitsbergen, Svalbard, Norway at 78° N, Svalbard reindeer (*Rangifer tarandus platyrhynchus*) and sibling voles (*Microtus levis*) are the only herbivorous mammals. The voles inhabit steep slopes associated with coastal bird cliffs [24] while the reindeer predominantly use inland valleys. Strong interspecific competition for forage resources between the reindeer and vole populations is therefore unlikely. Furthermore, reindeer and rodents share no predators or parasites that are likely to synchronize their dynamics.

We surveyed the populations annually in July–August in two adjacent locations on Nordenskiöld Land peninsula, Spitsbergen (see the electronic supplementary material, figure S1). In a small study area, we monitored the vole population size (1996–2007, *n* = 12 years), whereas monitoring of reindeer population size and the proportion of female reindeer with a calf occurred in neighbouring valleys (1995–2011, *n* = 17 years). Calves per female was chosen as the focal demographic parameter for reindeer as it is estimated with higher precision than population size, is known to be sensitive to environmental variation [14], and is positively related to the growth rate of the population (*r* = 0.59).

Daily precipitation and temperature data come from the meteorological station at the airport in the nearby settlement Longyearbyen (see the electronic supplementary material, figure S1). From these data, we calculated the amount of precipitation that fell at a temperature above 1°C, between 1 November and 30 April, as our measure of the extent of ROS. We used generalized linear models to investigate the impact of ROS and population size the previous summer on reindeer fertility and vole population sizes. In the analysis of the reindeer fertility data, we assumed a logit link function, and in the analysis of vole population sizes we assumed a log link function. Owing to overdispersion in the data in relation to the natural distributional assumptions the models were fitted using quasi-likelihood methods ([25], see the electronic supplementary material for details on data and analyses).

## 3. Results

**Figure 1.**
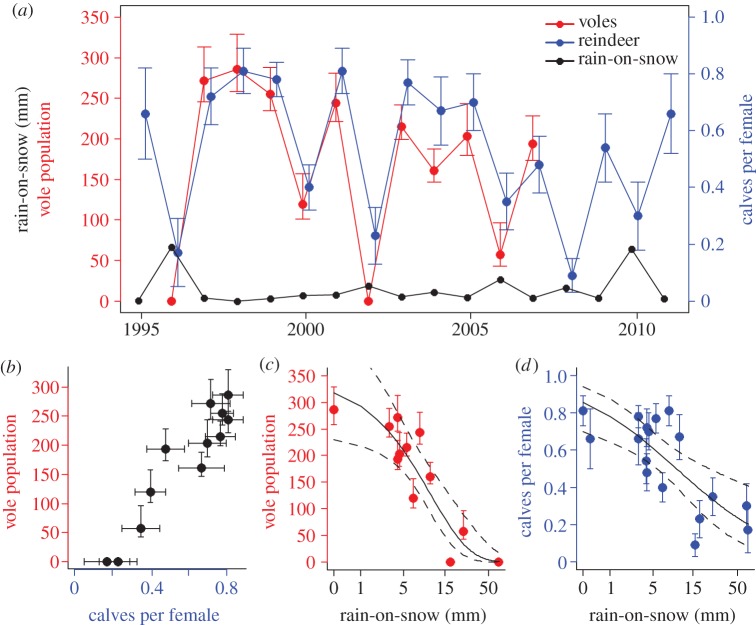
Annual estimates of sibling vole population sizes, calves per female Svalbard reindeer and ROS. (*a*) Time series of annual estimates (± 95% CI) of sibling vole population sizes (red colour, 1996–2007), Svalbard reindeer fecundities (calves per female (blue colour, 1995–2011) and ROS (black colour; mm, 1995–2011). (*b*) Estimated vole population sizes plotted against calves per female reindeer. (*c*) Vole population sizes plotted against ROS. (*d*) Calves per female reindeer plotted against ROS. Regression lines for the univariate relationships with 95% confidence envelopes (dashed lines) are given in (*c*) and (*d*) with ROS along the *x*-axis plotted on log(*x* + 1) scale.

Vole population sizes and calves per female reindeer varied extensively over the study period. The estimated vole population size fluctuated between 0 and 286 individuals with no evidence of population cycles, while calves per female reindeer varied between 0.16 and 0.80 (figure 1*a*). The fluctuations in vole population size and calves per female reindeer were highly congruent (figure 1*b*; *r* = 0.95, *p* < 0.001). This suggests that both the reindeer and vole population were responding to a common environmental stimulus in this high Arctic ecosystem.

ROS had a strong negative effect on both vole population sizes (figure 1*c* and table 1) and calves per female reindeer (figure 1*d* and table 1). The lowest observations of vole population sizes and calves per female reindeer were all associated with more than 15 mm ROS (figure 1*c,d*). When the effect of ROS was controlled for statistically there was little evidence for a negative effect of previous year's population size on vole population sizes (table 1), but a clear effect of previous year's reindeer population size on calves per female reindeer (table 1). The full models had high *R*^2^-values (table 1), however there was still evidence for additional unexplained covariance between the species in the residuals from these models (*r* = 0.75, *p* = 0.005).

**Table 1.** Parameter estimates (with 95% CI), QAICc and *R*^2^-values for GLMs for the impact of rain-on-snow (ROS) and previous year's population size (DD) on calves per female reindeer and vole population sizes. QAICc values for the best fit models are in bold.

response	model	parameter estimates (95% CI)				
		intercept	ROS (mm)	DD	QAICc	*R*^2^-values
calves per	intercept	0.28 (−0.2, 0.8)			62.8	
female^a^	ROS	1.8 (0.9, 2.7)	−0.75 (−1.18, −0.37)		42.2	0.57
	DD	3.8 (2.1, 5.7)		−0.010 (−0.015, −0.005)	40.0	0.39
	ROS + DD	3.7 (2.2, 5.2)	−0.46 (−0.84, −0.11)	−0.007 (−0.012, −0.002)	**36**.**7**	0.68
vole	intercept	5.1 (4.8, 5.4)			63.2	
population^b^	ROS	5.8 (5.5, 6.0)	−0.084 (−0.13, −0.05)		**25**.**5**	0.81
	DD	5.5 (4.7, 6.2)		−0.098 (−0.24, 0.07)	61.7	0.15
	ROS + DD	5.9 (5.5, 6.3)	−0.083 (−0.13, −0.05)	−0.036 (−0.12, 0.05)	29.5	0.83

^a^ROS fitted as log(ROS + 1) in the models.

^b^DD fitted as log(DD + 1) in the models.

## 4. Discussion

This study demonstrates a strong population dynamic synchrony between voles and reindeer in a high Arctic ecosystem that is predominantly due to the extent of joint climatic forcing. Ground ice caused by ROS events has previously been shown to affect reindeer [17], while there has been less evidence for an effect on high Arctic small rodents [3]. Our study is the first to show a strong impact on both ecosystem components in the same arctic ecosystem. Climate change is expected to increase the frequency of ROS events in large parts of the Arctic [22,26] and our results suggest that these extreme weather events might increasingly become a main determinant of the structure and functioning of arctic ecosystems.

After correcting for the effects of ROS and density dependence, there was still some evidence for residual correlation between the species. This suggests that our estimator of ROS does not capture all the climatic variation that impacts the two herbivores. A better understanding of the processes that generate ground ice as well as direct measures of ground ice might allow improved predictions. Alternatively, there might be additional climatic factors that affect the food quality and quantity available for both voles and reindeer in this ecosystem.

We are aware of only one previous study that has demonstrated climate-induced interspecific synchrony among arctic animals. Post & Forchhammer [27] found that populations of musk oxen (*Ovibos moschatus*) and caribou (*Rangifer tarandus*) were synchronized by regionalized weather patterns across Greenland. In that case, the two focal herbivores were both large ungulates with similar life histories. Thus, our study adds to the knowledge about the population dynamics of Arctic herbivores by providing a compelling demonstration that climate impacts can be integrated and expressed similarly in species with highly contrasting life histories.

There are two contexts of the high Arctic environment that might facilitate the extraordinary interspecific congruent responses. One is the extreme variability of the winter climate on Svalbard with frequent and profound ROS events [22], a pattern seen over extensive areas in the Arctic [22,26]. The other is the simplicity of the terrestrial food web at Svalbard, in particular without specialized predators that could drive multi-annual population cycles due to delayed density dependence [5]. It could be expected that populations with different and strong density dependence would be less prone to climate-induced interspecific synchrony [28]. Consequently, responses of wildlife populations to climate variability and change might indeed be more consistent and more predictable in the most extreme environments of the Polar regions, than terrestrial ecosystems elsewhere.
